# Secrets and Their Consequences in Heathcare: A Scoping Review of Worker Experiences

**DOI:** 10.1111/jan.16922

**Published:** 2025-03-30

**Authors:** Kiriaki Stewart, David Evans, Qunyan Xu, Nicholas Procter

**Affiliations:** ^1^ Clinical and Health Sciences University of South Australia Adelaide South Australia Australia; ^2^ Mental Health and Suicide Prevention Research and Education Group University of South Australia Adelaide South Australia Australia

**Keywords:** healthcare consumer, healthcare worker, private disclosure, secret, stigma

## Abstract

**Aims:**

To explore current knowledge of the nature of secrets healthcare consumers reveal to healthcare workers and the impact on the healthcare worker experience resulting from these private disclosures.

**Design:**

Scoping review.

**Data Sources:**

Eight electronic databases were searched for studies published between 1980 and 2025 that met the inclusion criteria.

**Review Methods:**

Arksey and O'Malley's five‐step methodological framework for scoping reviews guided this review. Petronio's Communication Privacy Management theory also informed the inclusion criteria.

**Results:**

A total of 11,812 records were identified, and 76 studies were included. Four types of secrets emerged: (1) Medical concerns, (2) Sexuality, (3) Abuse and (4) Life choices and decisions. Healthcare workers viewed the disclosure experience either as a privilege or a burden. Healthcare workers constructed strategies to avoid or facilitate disclosure and sought out trusted confidants for personal support throughout the experience. The review highlights the vulnerability of both the healthcare consumer and the healthcare worker, and the potential for negative health outcomes following a private disclosure.

**Conclusion:**

Healthcare consumers make private disclosures to healthcare workers. Further research is needed to construct evidence‐based clinical practice guidelines to support healthcare workers in facilitating and managing private disclosures.

**Impact:**

Healthcare consumers may disclose secrets of sexuality, medical concerns, abuse, and life decisions and choices to healthcare workers. Limited studies report on the impact of these difficult conversations. The findings of this review can inform healthcare practice and further research.

**Patient or Public Contribution:**

Not applicable.

*I've got a secret and I can't explain…*

*There are things I cannot tell…*

*This is getting very hard for me…*

(McClusky and Humphreys 1985)

## 1 Introduction

Every person has secrets that they do not wish to be commonly known. Highly sensitive secrets people ruminate upon can negatively affect physical and mental well‐being (Slepian et al. 2017).

In nursing and healthcare contexts, consumers are required to share personal information that may be private secrets when accessing services. Secrets healthcare consumers share with healthcare workers reported in literature can be grouped across four main types:

- *Abuse*, for example, child abuse (Hoffer 2017, 137; Pritchard 2007, 109), intimate partner violence (Hewitt et al. 2011, 320) or elder abuse (Afifi and Stueber 2009, 155; Ingersoll 2016, 133).
- *Sexuality*, including sexual orientation and sexual behaviour, for example, lesbian, gay, bisexual, transgender, intersex, queer or questioning, asexual, pansexual, polysexual, same‐sex attraction and other diverse identities (LGBTIQAP+) (Hughes 2008a, 2008b; Neville et al. 2015; Robinson 2016; Willis et al. 2016).
- *Medical concerns*, for example, eating disorders (Gilbert et al. 2012, 84); substance dependency, for example, alcohol dependency (Salladay 2012, 9); or a positive human immunodeficiency virus (HIV) status (Schwolsky 2011, 72).
- *Life choices and decisions*, for example, infidelity (Petronio and Sargent 2011, 261).

Sharing secrets has impact. Both the healthcare consumer and the healthcare worker can experience physical and mental distress (Petronio and Sargent 2011, 264). As the worker and consumer interact with each other, the healthcare worker's response to the secret may have collateral effects upon consumer well‐being. Negative responses increase healthcare consumer trauma. Sharing a secret with a trusted healthcare worker may provide relief from the burden of a secret; however, fear of a negative response can prevent a person from sharing sensitive personal details (Hughes 2008b, 17).

Conversations where healthcare consumers disclose secrets either expectedly or unexpectedly are challenging. Healthcare workers may seek support to respond appropriately to healthcare consumers with secrets and to regulate excessive attention attributed to the secret they are entrusted with. Where a person is not able to share their secret, for example, they do not have a trusted confidant or are legally bound to confidentiality, reducing constant thoughts of a secret can have positive health outcomes (Slepian et al. 2017, 28).

Despite healthcare consumers being known to disclose potentially confronting secrets to healthcare workers, little is reported of the nature of these secrets, or the health worker experience and response to these difficult conversations in the literature. This review aims to address this omission. It aims to add valuable knowledge, document current practices and guide future ways of working.

## 2 The Review

### 2.1 Aim/s

The aim of this scoping review was to investigate and document the nature of secrets healthcare consumers reveal to healthcare workers, and the healthcare workers' experience and response to private disclosures made to them by healthcare consumers.

### 2.2 Design

The conceptual basis of this scoping review was broadly informed by Communication Privacy Management (CPM), a disclosure theory by Petronio (2002). CPM is founded on the premise that an ‘owner’ of a secret may share their secret with a confidant who then becomes a ‘co‐owner’ of the secret (Petronio and Sargent 2011, 256). The need to keep a secret private and the need to share a secret causes tension (Petronio and Durham 2008, 311–312). Owners may stipulate privacy rules and attempt to dictate boundaries for their secret that the co‐owner must not violate. Breaking these rules, for example, sharing the secret with another person against the owner's demands creates turbulence (Petronio and Sargent 2011, 263). The nature of the secret and the process of sharing a secret are both key to understanding the experience (Petronio 2004, 195). The perspective of the discloser who shares the secret is equally as important as that of the recipient who receives the secret, and both warrant exploration (Petronio 2002, 16). CPM continues to evolve and has been used for nursing research (Petronio and Sargent 2011).

The scoping review technique followed Arksey and O'Malley's five stages for scoping reviews (Arksey and O'Malley 2005, 22). A scoping review was deemed appropriate as they are a useful medium to map the breadth and depth of a little‐known phenomenon (Arksey and O'Malley 2005; Levac et al. 2010, 2). The ‘Preferred Reporting Items for Systematic reviews and Meta‐Analyses extension for Scoping Reviews (PRISMA‐ScR) Checklist’ (see Appendix S1) was also used (Tricco et al. 2018), including a review protocol which was not published.

For the purposes of this scoping review, a ‘secret’ is personal information a healthcare consumer perceives as highly sensitive and private. A ‘discloser’ is a healthcare consumer who revealed confidential secret information. A ‘recipient’ is a healthcare worker who received a secret from a healthcare consumer. A ‘private disclosure’ is the revelation of a secret to a healthcare worker by a healthcare consumer.

The research questions are:

1. What is the nature of private disclosures made by healthcare consumers to healthcare workers?
2. What do healthcare workers think, observe or experience within themselves in response to a private disclosure?

### 2.3 Search Methods

Searches were performed in the Embase, Emcare, Google Scholar, MEDLINE (Ovid), ProQuest Central, APA PsycINFO, SCOPUS and Web of Science databases, using the search equation (context terms) AND ((participant1 discloser terms) OR (participant2 recipient terms)) AND (concept terms). Relevant search terms/MeSH terms were developed in consultation with an expert librarian. Articles were restricted to English publications. The resulting searches are included in Appendix S2, and a flow chart in Figure 1. Reference lists of identified articles were also inspected. Grey literature searches were not formally performed, as preliminary searches did not identify any relevant studies.

Primary research studies meeting the following eligibility criteria were included in the review:

#### 2.3.1 Participants

Studies that focused on the two groups of participants, namely, the discloser and the recipient of the private disclosure, were included as follows:

*Healthcare consumer* (HC) of an acute healthcare service, residential care or community healthcare service, aged 18 years and above, who disclosed a secret to a healthcare worker.

*Healthcare worker* (HW) employed to provide a healthcare service, including: (1) Medical Officers (MOs), (2) Registered Nurses (RNs), (3) Enrolled Nurses (ENs), (4) Allied health staff and (5) Personal care workers (PCWs).

This review was concerned with the experience of staff who had not received formal training in psychology or counselling, therefore psychology or counselling HWs were excluded in the definition of the HW population.

#### 2.3.2 Concept

The concept was a private disclosure made by an HC to an HW. Inclusion criteria included: (1) the nature of a secret disclosed by an HC to an HW; and (2) what HWs think, observe or experience within themselves at the time of a private disclosure.

#### 2.3.3 Context

The context was any healthcare setting and included: (1) residential care, (2) acute care and (3) community care.

The search results were imported to Endnote for duplicate removal. Due to the large volume of search results, articles were firstly scanned against the inclusion criteria by title, and irrelevant articles were excluded. Articles were then screened against the eligibility criteria by abstract, and finally by full text. Screening was largely completed by Author 1. A handful of articles were inspected within the authorship team to validate this process in the initial stages. 5% of articles were screened by abstract in duplicate amongst three reviewers (KS, AX and KR), and 75% of articles were screened by full text in duplicate by two authors (KS and AX), with 95% inter‐rater reliability. Any discrepancies were discussed with a fourth author until consensus was reached.

### 2.4 Data Extraction

A Data‐charting form was constructed to extract study design, objectives, participant descriptions and numbers, concept (the secrets) and context data in an Excel spreadsheet. The team piloted and calibrated this form prior to use. Author 1 charted data with continued group discussion and review of the key study characteristics (Tricco et al. 2018).

### 2.5 Synthesis

Data relating to the review questions were analysed using qualitative content analysis. To address research question 1, data types and subtypes were organised by Participants, Concept and Context. Extensive group discussion revisited data types and subtypes from the background search for consistency. Data were analysed to extend beyond merely counting instances of secret types to exploration and interpretation of the underlying significance of the disclosure (Hsieh and Shannon 2005, 1284). To address research question 2, data were firstly searched for frequency of occurrences of the categories used in the data extraction tool. Patterns that emerged were explored and contrasted for context and meaning. These patterns were summarised and synthesised in both tabular and narrative form. The optional step of a critical appraisal of articles was not undertaken (Tricco et al. 2018).

## 3 Results

**FIGURE 1:**
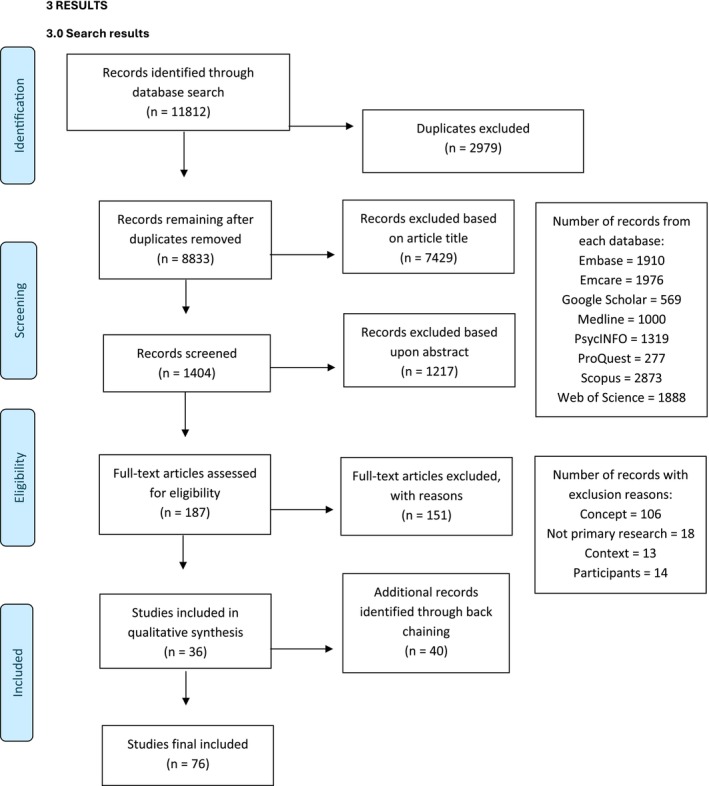
Search results.

### 3.1 Search Results

#### 3.1.1 Characteristics of the Included Studies

Seventy‐six studies included in the review were published between 1988 and 2025. Thirty‐six studies used a qualitative design, 35 studies used a quantitative design and five used a mixed‐methods design. Twenty‐three countries were represented in the studies, including the United States of America (US), Malaysia and the United Kingdom (UK).

##### 3.1.1.1 Context

Three contexts were represented. Forty‐one studies reported on data collected in community care, 23 studies reported on data collected in acute care, one study reported on data collected in a residential setting and 31 studies did not specify the specific healthcare context.

Acute healthcare contexts included hospitals, emergency departments, intensive care departments, acute care departments, thalassaemia wards, internal medicine departments and general hospital services. Community healthcare contexts included gynaecology clinics, urologist clinics, outpatients, pathology outpatients, community clinics, primary care clinics, sexual health clinics, family practice clinics, general practitioner clinics, antenatal care clinics, optometrists, dentist clinics, pharmacies and ambulances. A residential housing project represented a residential healthcare context. No studies were conducted in the context of residential aged care.

##### 3.1.1.2 Participants

HCs were participants in 68 studies and HWs were represented in 10 studies. HCs included persons from the LGBTIQ+ community, for example, men who have sex with men (MSM); persons living with a medical condition, for example, a positive HIV status; HCs of acute or community healthcare services, for example, acute hospital surgical wards, palliative care, gynaecology clinics, pharmacies, dentist services and ambulance services; women who self‐managed abortion; and HCs where the health service was not specifically reported. HWs included nurses, for example, Nurse Practitioners (NPs), nurses; medical professionals, for example, doctors, General Practitioners (GPs) and physician assistants (PAs); and other health professionals, including social workers (SWs), dentists, pharmacists and midwives. Demographics pertaining to study method, country, setting and participants are included in the summary of included studies in Appendix S3.

### 3.2 The Secrets

Data were extracted from 76 studies. Four types of secrets emerged: (1) Sexuality, (2) Medical concerns, (3) Abuse and (4) Life choices and decisions. See Appendix S3 for a summary of included studies.

#### 3.2.1 Sexuality

Sexuality is the most common secret, with 49 studies reporting secrets pertaining to sexuality. *Sexual orientation*, including lesbian (L), gay (G), bisexual (B), queer(Q), transgender (T), sexual and gender minority (SGM) or gender non‐conforming (GNC), and *Sexual behaviour*, including women who date women (WWDW) or MSM, are reported (Algarin et al. 2022; Agenor et al. 2015; Barbara et al. 2001; Bernstein et al. 2008; Bjorkman and Malterud 2009; Boehmer and Case 2004; Cant 2006; Cao et al. 2021; Chaudhary et al. 2022; Cochran and Mays 1988; Coleman et al. 2017; Currin and Hubach 2017; Doran et al. 2018; Durso and Meyer 2013; Eliason and Schope 2001; Fay et al. 2011; Fish et al. 2019; Hinrichs et al. 2018; Hitchcock and Wilson 1992; Hudak 2015; Jansen et al. 2024; Lane et al. 2008; Lasater et al. 2019; Lee et al. 2020; Lewis et al. 2019; Kamen et al. 2015; Macapagal et al. 2016; Metheny and Stephenson 2016; Mimiaga et al. 2007; Mosack et al. 2013; Nadler et al. 2021; Petroll and Mitchell 2015; Petroll and Mosack 2011; Raifman et al. 2017; Rank et al. 2012; Risher et al. 2013; Rose et al. 2016; Rounds et al. 2013; Ruiseñor‐Escudero et al. 2019; Shires et al. 2023; Singh et al. 2018; Smith and Turell 2017; Stablein et al. 2015; Thompson 2016; Wang et al. 2024; Wells‐Prado et al. 2022; Wiginton et al. 2021; White and Dull 1998; Yang et al. 2019).

Sexuality is highly personal and potentially stigmatising. Whilst a study in 1998 reported that 90% of lesbians in the US disclose their sexual orientation to their primary health providers (White and Dull 1998, 96), most studies report low numbers of HCs disclosing their sexuality to HWs. Only a third of black lesbian or bisexual participants in the United States disclosed their sexuality to HWs (Cochran and Mays 1988, 616). HCs reported experiencing intense anxiety, embarrassment, shame and awkwardness when they disclosed their sexuality to HWs. A participant described preceding anxiety of disclosing their secret of lesbian orientation:

I always have this knot or a jump in my chest… because there's always the risk of seeing the person react even if they say: ‘Oh, its fine’. But seeing something on their face. And so I experience the same thing with the providers (Agenor et al. 2015, 724).

Transgender participants in particular experienced stigma. A transgender person shared feeling dismissed when disclosing he was a man during an uncomfortable gynaecological exam:

… confided to the provider that he ‘felt like a guy’, the female provider responded: ‘I used to want to be a guy too because I thought it was really unfair that we have to have these periods and stuff. You'll get over it’ (Rounds et al. 2013, 105).

Similarly, transgender participants described their experiences of accessing gender‐specific medications or products at a pharmacy as ‘excruciating’, ‘probably the worst experience I've had’ and ‘humiliating’ (Chaudhary et al. 2022, 4). Another transgender participant recounted she was audibly misgendered in a public space and classified as ‘he/she?’ (Smith and Turell 2017, 646). A TGNC participant recounted a negative experience:

I visited a doctor at one point that almost ran out of the room when he got a sense I was different somehow… you could just smell the fear on him (Hinrichs et al. 2018, 78).

A lesbian participant felt awkward when disclosing her secret of bisexuality ‘At that time, I was actively dating a woman. I kind of said that, and her response was very… it was awkward’ (Agenor et al. 2015, 725). Another lesbian participant experienced her partner not being recognised as family:

… a nurse tried to find a family member of mine to explain my condition… the nurse was unwilling to explain my condition to my wife… I had already told the healthcare staff that… she was my wife… They all gave me a look like…What is same‐sex marriage? (Wang et al. 2024, 7).

A gay participant experienced increased shame whilst seeking healthcare for a sexually transmitted infection:

I got the clap a little while ago and they interrogated me: ‘Well this is no good – you're not doing safer sex, are you?’ – I've got enough guilt on my plate without them laying it on (Cant 2006, 14).

Participants also expressed concern about involuntary disclosure when completing healthcare paperwork prior to appointments, finding it ‘a little bit scary’ (Stablein et al. 2015, 233). A transgender participant described an unsafe disclosure:

I was in the waiting room, and two nurses decided to discuss me. However, they failed to realise that they had the button for the PA system on. The whole damn waiting room and clinic could hear what they were discussing… It was bad (Thompson 2016, 210).

Stigma and fear of disclosure of same‐sex intimacy is also related to cultural context. MSM in Swaziland reported psychological fear when seeking healthcare services along with the experience of stigma (Risher et al. 2013). HCs in Sub‐Saharan Africa who disclosed they were MSM to an HW were more likely to experience fear in seeking out healthcare services and more likely to avoid healthcare services (Wiginton et al. 2021, 5). This was consistent for MSM participants in Malawi, Namibia and Botswana (Fay et al. 2011, 1088), and South Africa (Lane et al. 2008, 3).

The disclosure of sexuality presented a challenge for an HC to voice his genuine feelings about healthcare. A participant who disclosed intimacy with men, found it ‘incredibly difficult’ to convey his fear of rectal examination pain when the HW joked ‘Well somebody like you, it won't make a lot of difference to somebody like you’ (Doran et al. 2018, 5).

The experience of disclosing sexual behaviour to an HW is also related to seeking out health services for a specific healthcare need. HCs in Togo who disclosed they engaged in same‐sex practices to an HW were more likely to disclose due to a healthcare need, for example, living with HIV or having experienced physical abuse (Ruiseñor‐Escudero et al. 2019). HCs reported disclosure was more likely to occur when an HW assumed they were heterosexual (Kamen et al. 2015, 47). Also, sexual orientation was more likely to be disclosed than sexual behaviour, with bisexual men less likely to disclose MSM to an HW (Bernstein et al. 2008; Durso and Meyer 2013, 35).

To this end, the literature shows it is common for HCs to have secrets of sexual orientation or sexual behaviour. The disclosure of sexuality compounded HC vulnerability and is associated with an intense fear of a negative HW response.

#### 3.2.2 Medical Concerns

Seventeen studies reporting secrets pertaining to medical concerns. This type includes three sub‐types: (1) Complementary and alternative medicine (CAM) use, (2) HIV status and (3) Substance dependency.

##### 3.2.2.1 CAM Use

Eleven studies identified that HCs regarded the use of CAM as a secret from HWs who specialised in mainstream Western medicine (Ait Gacem et al. 2024; Ashikaga et al. 2002; Chang et al. 2013; Farooqui et al. 2012; Ismail et al. 2018; Jin et al. 2022; Johny et al. 2017; Kelak et al. 2018; McCrea and Pritchard 2011; Oldendick et al. 2000; Simundic et al. 2018). These studies reported a wide range of CAM, which included biologically natural or herbal based therapies (i.e., vitamins), traditional ethnic medicines (i.e., traditional Chinese medicine or Native American healing), spiritual therapies, homeopathy, ‘mind–body’ therapies (i.e., meditation), dietary programmes (e.g., commercial weight loss programmes), self‐help groups and manipulative based therapies such as massage.

These studies reveal HCs were hesitant to disclose CAM use that they initiated in secrecy, either without the knowledge or against the advice of HWs. HCs feared that HWs would respond negatively, and reprimand or reject them. Participants made comments about HWs such as ‘won't be happy’, ‘would be angry with me if I told him that I am still using it’ ‘stop the hospital medicine’ and ‘not entertain me’ (Farooqui et al. 2012, 254).

One participant described fear of being reprimanded when considering disclosing CAM use:

… I am afraid of being scolded … because some of the healthcare professionals will reject it … and also scold you … ‘if you are so good at this, you can just treat yourself’ (Chang et al. 2013, 6).

This was reality for a Chinese immigrant with Hepatitis B when disclosing her choice to continue with CAM instead of conventional medicine to Australian doctors:

The doctors were shocked, ‘How dare you make this decision [of not taking medication] without discussing it with us?’ (Jin et al. 2022, 972).

The use of CAM in opposition to actual or perceived advice of mainstream Western medicine HWs was an unexpected HC secret represented in the literature. The cultural context for most of these studies was Asia, for example, Malaysia (Farooqui et al. 2012; Ismail et al. 2018; Johny et al. 2017; Kelak et al. 2018) and Taiwan (Chang et al. 2013). The dynamic between a lack of HC trust in Western medicine and the significance of traditional healthcare practices is important in understanding why this is a sensitive secret for HCs. Fear of displeasing an HW also intensified secrecy surrounding CAM use.

##### 3.2.2.2 HIV

Five studies identified HIV (Arrey et al. 2015; Brinsdon et al. 2017; Charbonneau 1999; Turton and Naidoo 2014; Wong and Wong 2006) status as an HCC secret. All five studies associate stigma with positive HIV status.

Hesitancy to disclose positive HIV status to an HW was influenced by observed HW attitudes and fear of discrimination. A participant seeking fertility treatment encountered her experience:

I was refused the opportunity to become pregnant ‘in vitro’ in a fertility clinic because I am HIV positive. I was very disappointed because I wanted to become a mother. I left that clinic and went to another clinic that agreed to make my dream of becoming a mother come true (Arrey et al. 2015, 13).

Surprisingly, the most common healthcare context for studies investigating HIV status disclosure was dentist clinics (Arrey et al. 2015; Brinsdon et al. 2017; Charbonneau 1999; Turton and Naidoo 2014). Participants who disclosed their HIV status and were subsequently allocated the last appointment of the day interpreted this wait as discrimination. Other healthcare contexts where participants felt discriminated against when they disclosed their HIV status included hospitals (Brinsdon et al. 2017; Wong and Wong 2006), pharmacies (Arrey et al. 2015), general practitioner clinics (Arrey et al. 2015), optometry clinics (Brinsdon et al. 2017), sexual health clinics (ref) and non‐government organisations that provided care for HIV positive persons (Wong and Wong 2006). A participant who chose to delay disclosing their HIV status until they presented for surgery reported feeling discriminated against by their Otolaryngologist:

When I went into the ENT (ear, nose and throat) clinic, the nurse asked me a few times if I had infectious disease. Actually I could feel the discrimination because when I was in the clinic, I wasn't the last one going in. But they put me into the last appointment … Then the doctor started to ask me why I didn't inform them about this disease [HIV]. The doctor said what if he injured himself during the operation and I infected him with HIV, then I would ruin his family. He said he has a wife and children and he is the family support. At that time I felt very unhappy because of what he said, but what can I do? (Wong and Wong 2006, 102).

These studies drew attention to the increased vulnerability of a positive HIV or Hepatitis C status and persistent stigma within the healthcare context. HIV Hepatitis C status is reported as a secret an HC may disclose to an HW.

##### 3.2.2.3 Substance Dependency

One study reported on substance dependency, namely non‐compliance with drug rehabilitation treatment (Tiderington et al. 2013). This study investigated the experience of the HW. A participant acknowledged HC reluctance of disclosing substance use was related to the risk of eviction, as sobriety was a condition of their accommodation (Tiderington et al. 2013, 74).

These studies demonstrate CAM use, HIV or Hepatitis C status, and substance dependency were medical concerns viewed as private and sensitive enough by HCs to be held secret. Stigma surrounding medical concerns and fear of negative consequences compounded the vulnerability of HCs. Medical concerns were the second most common HC secret disclosed to an HW reported in the literature.

#### 3.2.3 Abuse

Six studies raised abuse as a secret that an HC disclosed to an HW. Five studies reported on interpersonal violence (IPV) (Alvarez et al. 2018; Anguzu et al. 2022; Morris et al. 2025; Papadakaki et al. 2014; Willie et al. 2023) and one study on a history of sexual trauma within a military context (MST) (Bergman et al. 2019). All six studies drew attention to the vulnerability of this population.

Context is important in understanding the sensitivity of these secrets, as they are reported in contexts where society may not always be supportive of victims (Papadakaki et al. 2014, 375).

Disclosure of abuse is dependent on trust. IPV disclosure is associated with a ‘trustworthy and confidential Patient‐Provider relationship’ (Morris et al. 2025, 496).

Abuse is reported in the literature as an HC secret that may be disclosed to an HW. Victim shame and guilt is associated with hesitancy of disclosure, whilst HCC‐HCW trust is highlighted as ‘essential’ for disclosure (Morris et al. 2025, 490).

#### 3.2.4 Life Choices and Decisions

Four studies reported secrets related to life choices and decisions, including self‐managed abortion (Fuentes et al. 2020; Keefe‐Oates et al. 2022), infidelity (Petronio and Sargent 2011) and the decision to secretly give children up for adoption (Hersh 2015).

Women in Texas only revealed they had attempted self‐managed abortion to HW when their method was unsuccessful, putting their health at risk (Fuentes et al. 2020). Women who self‐managed abortion post 13 weeks gestation in Argentina were fearful of seeking healthcare services as disclosure risked legal consequences. It is important to acknowledge consequential differences of self‐managed abortion disclosure between contexts where abortion is illegal and where abortion is legal and viewed as a human right.

A participant who was 18 weeks pregnant described her negative experience when disclosing she was considering abortion to her doctor:

On top of not helping me, [the doctor] made me more scared… saying ‘no, if you are going to do it you could go to prison’… (Keefe‐Oates et al. 2022, 6).

A participant reported an HC confided he feared his affair would be revealed to his wife if he disclosed it to his doctor (Petronio and Sargent 2011, 261). Another participant understood the need of a dying patient to confess they planned to secretly arrange for their children to be given away (Hersh 2015, 61). HCs harbouring these secrets feared disclosure would instigate negative and legal consequences.

Medical concerns, sexuality, life choices and decisions, and abuse are reported in the literature as secrets that may be disclosed by HCs to HWs. The included studies demonstrate a relationship between the secret and HC vulnerability. HCs expressed a fear of experiencing negative reactions, repercussions and personal emotions when disclosing their secrets. This fear was substantiated in numerous participant contributions. HW contributions validate the complexity of these secrets.

### 3.3 The HCW Experience And/or Response

Data pertaining to the HW experience and/or response to receiving a private disclosure from an HC were extracted from nine studies (Alvarez et al. 2018; Anguzu et al. 2022; Bergman et al. 2019; Hersh 2015; Nadler et al. 2021; Papadakaki et al. 2014; Petronio and Sargent 2011; Tiderington et al. 2013; Willie et al. 2023). Differences were reported with regard to what HWs thought, felt or experienced within themselves in response to receiving an HC secret, and these were reflected in the different strategies HCs employed in managing these disclosures. See Appendix S4 for a summary of included studies.

#### 3.3.1 The Health Worker Experience

The studies also demonstrated how HWs understood a unique relationship was forged between themselves and HCs due to the disclosure of a secret. HWs viewed the resulting relationship as either a burden or a privilege of their role.

##### 3.3.1.1 The Burden

Seven studies reported that HWs viewed the disclosure experience as a burden (Alvarez et al. 2018; Anguzu et al. 2022; Bergman et al. 2019; Nadler et al. 2021; Papadakaki et al. 2014; Petronio and Sargent 2011; Willie et al. 2023). Participants described discomfort, feelings of inadequacy, a lack of time, a lack of support and potential risk in managing private disclosures.

Nurses providing care to HCs in intensive care units, emergency departments and oncology units viewed disclosure experiences as a burden. One participant described the tension between their role in providing person‐centred care and their personal feelings with regard to the burden of the extra workload a disclosure creates:

You don't want to hear, you just don't want to hear … you have your own problems, too. You are just working, this is a job … [so in order to do the job she said] you know, just tell me what the problem is and let's get out of here (Petronio and Sargent 2011, 260).

Some participants felt they were not adequately prepared or supported to manage private disclosure. An NP screening for IPV in an antenatal clinic described their discomfort:

I am not prepared… at all… I cringe every time (Alvarez et al. 2018, 151).

A gynaecologist reported the burden of inadequacy when discussing sexuality with LGBTQ+ patients:

I honestly don't feel that I have gotten really good about asking those questions … (Nadler et al. 2021, 905).

Other participants felt they did not have time to provide adequate care to HCs post a private disclosure. A participant who performed pap smears for women with a history of MST stated:

It always seems to take like three or four times as long to get something done. It took me almost a year to get her Pap smear done and the challenge for me is I'm someone that likes to get things done now (Bergman et al. 2019, 327).

A doctor in Greece reported that providing IPV‐related care created a burden for their busy schedule:

I have so many patients waiting outside my office every day. Can you imagine asking all these patients about their relationship with their partner? I would need fairly more time for each patient (Papadakaki et al. 2014, 374).

Participants expressed a lack of support in managing private disclosures. A doctor in Greece expressed their frustration at this lack of support for victims of IPV:

I once contacted a community mental health center to request assistance for a high‐risk case. They told me that they could only accept an application from the abused woman herself and by no person other than the victim… I realized I could not do anything for her and let her go home (Papadakaki et al. 2014, 375).

Participants were also concerned that the HC secret could put their own safety at risk. A doctor shared this fear:

What if someone identifies the patient and spreads the information to her family… Who will guarantee my safety then? (Papadakaki et al. 2014, 375).

The literature demonstrates that HWs view private disclosures as a burden resulting in feelings of inadequacy and discomfort. The way disclosures impact an HW's ability to provide adequate care can negatively affect well‐being.

##### 3.3.1.2 The Privilege

In contrast, participants in six studies viewed the experience of receiving a private disclosure from an HC as a ‘good’ part of their role (Alvarez et al. 2018; Anguzu et al. 2022; Hersh 2015; Petronio and Sargent 2011; Tiderington et al. 2013; Willie et al. 2023). Participants understood they were recipients of privileged personal information required to provide care to HCs. An RN reflected on the information HCs share during emergency setting admission screenings:

They go into really personal details … And they tell you, they will tell you (Petronio and Sargent 2011, 259).

Other participants described the privilege of receiving a private disclosure from an HC as a ‘gift’ and ‘special’ (Hersh 2015, 77, 88). A nurse described their interactions with an HC at the end‐of‐life stage:

It begins as an encounter the nurse has with the patient, and slowly becomes a connection that goes beyond time and space. You are fully focused on the patient, time seems to stop (Hersh 2015, 88).

All participants recognised that receiving private disclosures from HCs was part of their role to some extent. Differences were present with relation to the depth at which the HW was willing to connect with the HC in the HW–HC relationship. This influenced the strategies HW used to manage private disclosures.

#### 3.3.2 HW Strategies

HW management of private disclosures made to them by HCs was reported in all nine studies. This included avoidance to limit the opportunity of disclosure, active facilitation to provide the opportunity of disclosure and neutrality where disclosures were neither encouraged nor discouraged. HWs also developed strategies to support themselves to be able to manage professional and personal needs related to receiving a private disclosure.

##### 3.3.2.1 Avoidance

Eight studies reported HWs actively avoided HC disclosures (Alvarez et al. 2018; Anguzu et al. 2022; Bergman et al. 2019; Nadler et al. 2021; Papadakaki et al. 2014; Petronio and Sargent 2011; Tiderington et al. 2013; Willie et al. 2023). Strategies included ignoring the secret, referring the case to another colleague, department or profession, and physically avoiding the HC to limit opportunity for disclosure.

Participants managing a housing project for HCs with a history of drug dependency avoided bringing up recent drug use and ignored the ‘open secret’ (Tiderington et al. 2013, 74). This protected the HC from possible eviction, gave time for trust to develop and maximised the possibility of aiding sobriety once the HC was open to receiving this assistance.

Conversely, a doctor in Greece describes how they treated physical injury, ignoring the ‘open secret’ of IPV as they did not perceive this their role to manage:

A woman once came to my practice with a broken leg… she confessed it was not an accident, but her husband lost control… I treated her wound, prescribed the necessary medication, explained to her how to care for the wound, and asked her to visit me again to monitor the healing process… this was my only duty as a doctor (Papadakaki et al. 2014, 374).

Participants also used the strategy of referring cases on to avoid managing private disclosures. The studies report this was due to a perceived lack of skill in managing the content of the disclosure, or to ensure the healthcare was provided by an HW with experience in managing the specific needs related to the disclosure (Bergman et al. 2019, 328). For example, a participant shared their stance of ‘handing off’ HCs with a history of MST when providing healthcare to women veterans:

I am not, myself, going to do the gynaecological exam on an individual who's got military sexual trauma when we have nurse practitioners who are fantastic (Bergman et al. 2019, 328).

Yet another participant shared their avoidance strategy of avoiding roles where they may be in the frontline to receive a private disclosure (Petronio and Sargent 2011, 260). These studies confirm that HWs use a variety of strategies to avoid receiving a private disclosure from an HC.

##### 3.3.2.2 Facilitation

All nine studies reported on the strategies HWs used to facilitate receiving a private disclosure from an HC. These included straightforward questioning for required information, fostering an HW‐HC relationship to build trust, creating a safe space and purposely adopting a role where a disclosure was likely to occur.

Participants also acknowledged they were privy to confidential personal details collected in the process of receiving healthcare services. A urologist stated that asking questions specifically eliciting personal disclosures was routine for them:

Working in urology, we ask a lot of sensitive questions in general… from a urology standpoint… about sexual orientation, gender identity (Nadler et al. 2021, 906).

Participants actively nurtured a relationship with HCs (Alvarez et al. 2018; Anguzu et al. 2022; Bergman et al. 2019; Hersh 2015; Papadakaki et al. 2014; Petronio and Sargent 2011; Tiderington et al. 2013; Willie et al. 2023). Participants commented on the importance of patience in establishing the HW‐HC relationship to develop trust (Bergman et al. 2019; Tiderington et al. 2013).

A nurse expressed the importance of this relationship as an antecedent to disclosure:

It depends on your relationship with them [patients]… If they feel that you're a safe person to them, that you're not judging them, that you have the time for them, you don't have a rushed manner with them, that you're there (Petronio and Sargent 2011, 259–260).

HWs managing a housing project for persons with drug dependency noted drug use was an ‘open secret’ not yet openly acknowledged. Successful intervention was dependent on the trust relationship HWs developed with HCs (Tiderington et al. 2013, 73). A provider summarised:

We give second, third, fourth, fifth, sixth chances… You have to wait for them – the only thing you can do is encourage and support… I mean I can't say, ‘You need to stop using.’ … We put you in apartment and we're providing support, and if you want to stop using – I can encourage you everyday, and when you're ready I – I have the resources that you need to stop using. I can make those resources available to you (Tiderington et al. 2013, 74).

Similarly, a participant shared how they exercised patience to develop an intimate trust relationship with a challenging HC with a history of MST:

I was very patient with her [the patient] because I was trying to form a relationship … it was probably from her MST that we got all this guarded and kind of gruff behavior (Bergman et al. 2019, 329).

Four studies also reported HWs purposely created a safe space for an HCs to disclose a sensitive secret to them (Alvarez et al. 2018; Bergman et al. 2019; Petronio and Sargent 2011; Tiderington et al. 2013). Participants shared how they used triggers to know when ‘meaningful interactions’ were needed, including noting when an HC was labelled as ‘difficult’ by other HWs. A palliative care nurse articulated how this signalled a shift change to position themselves as the ‘right nurse’ at the ‘right time’:

When I heard this I knew the patient had not yet had a meaningful exchange with a nurse. It was time for me to do damage control (Hersh 2015, 63).

An NP screening for IPV shared a deliberate strategy:

… we screen every new patient and once a year… We don't allow partners back into the room with the patient (Alvarez et al. 2018).

These studies demonstrate HWs use a variety of strategies to facilitate private disclosures. The overarching sentiment that emerged was the HW‐HC relationship. Trust was revealed as a precursor for disclosure to occur, as well as a distinctive result of disclosure. The studies also underscore strategies are guided by the HW's experience and influenced by their personal view of their abilities and professional role.

##### 3.3.2.3 Neutrality

Alternatively, participants managed the potential for private disclosures with neutrality. At times, this was to avoid HC offence or HW discomfort. A participant shared their neutral stance:

I don't have a good way of asking about gender identity. Usually that will be something the patient will bring up to me. I'm sure there's times where a patient might feel uncomfortable because I haven't asked (Nadler et al. 2021, 906).

These studies show that HWs are aware private disclosures may occur when interacting with HCs. They also show that HWs have experienced this phenomenon and have developed a range of active or inactive strategies to manipulate the possibility of private disclosures.

#### 3.3.3 Professional and Personal Support

All nine studies discussed professional or personal needs HWs experienced with respect to receiving a private disclosure from an HC (Alvarez et al. 2018; Anguzu et al. 2022; Bergman et al. 2019; Hersh 2015; Nadler et al. 2021; Papadakaki et al. 2014; Petronio and Sargent 2011; Tiderington et al. 2013; Willie et al. 2023). HCWs developed strategies to support these needs. A doctor created shortcuts to streamline entering gender and sexuality data, reducing time navigating an inadequate electronic health record system (Nadler et al. 2021, 907). Other participants added buffers in appointment schedules, allowing time to appropriately respond to a potential disclosure (Bergman et al. 2019, 328).

Participants in all nine studies acknowledged experiencing a range of emotions when receiving a sensitive disclosure from an HC. Participants stated they managed personal needs by seeking out confidential emotional support from colleagues (Petronio and Sargent 2011, 263). A Palliative care nurse described the criteria for identifying a trusted confidant with whom to debrief:

I look for nurses with similar nursing philosophies, who have felt attachment to a patient, who also seek meaningful patient exchanges (Hersh 2015, 65).

Through trial and error, HWs found ways to manage professional or personal needs. Professional needs mostly related to functional resources and time. Personal needs mostly related to seeking support with emotional well‐being.

Data extracted from the nine included studies show that there is a range of what HWs feel, observe and experience in response to a private disclosure made to them by a HC. Previous experience and confidence in personal abilities influenced strategies used prior to a private disclosure, for example, active avoidance, active facilitation or a neutral approach. HWs experienced a range of professional or personal needs arising from disclosure and used personally constructed strategies to dedicate time to HCs where needed or seek out emotional support from trusted colleagues.

## 4 Discussion

The findings from this scoping review show HCs hold private secrets that are sometimes disclosed to HWs. The broad categories of the secrets identified, namely sexuality, medical concerns, abuse, and life choices and decisions, are consistent with the background literature search.

This review highlights the deeply sensitive, potentially stigmatising and severe consequential nature of personal secrets, as well as the vulnerable position of the HC. Trust is associated with whether the secret is disclosed to an HW or not. Secrets are influenced by culture, epoch, societal changes and political stance; for example, disclosing termination of pregnancy can result in legal repercussions in some countries (Keefe‐Oates et al. 2022; Fuentes et al. 2020).

Most articles reported on the secret of sexuality. Sexual orientation and sexual behaviour beyond heterosexuality remain illegal in conservative societies and disclosure consequences include repeated physical, mental, and sexual abuse, imprisonment, stigma and shame (Fay et al. 2011, 1088). Stigma and shame also persist in less conservative societies (Chaudhary et al. 2022).

Non‐disclosure was associated with an increased risk of detrimental health consequences. For example, not disclosing MSM was related to decreased screening for sexually transmitted diseases and related vaccinations (Rank et al. 2012, 5755). Not disclosing being transgender resulted in a lack of access to required healthcare resources (Chaudhary et al. 2022, 4). Not disclosing self‐managed abortion increased the risk of infertility and death (Keefe‐Oates et al. 2022, 6). Involuntary disclosure is infrequently discussed, yet it is expected that HCs yield personal information when accessing healthcare services. Involuntary disclosure raises the issue of a person's right to self‐determination, a contemporary debate in residential aged care (Moilanen et al. 2021).

This review also brings attention to tension between providing person‐centred care to vulnerable HCs and potential HW vulnerability in receiving a private disclosure. Person‐centred care is, in this context, about ensuring personal stories and lives are revealed to guide care preferences (eds Procter et al. 2022). HCWs found themselves vulnerable to feelings of stress with a lack of time to provide sensitive care to women who had experienced MST. HWs constructed strategies to mediate this vulnerability on their own, for example, referring HCs to another HW or health service (Bergman et al. 2019, 328), labelling HCs as ‘difficult’ (Hersh 2015, 63), or providing physical medical care and avoiding the ‘open secret’ of IPV (Papadakaki et al. 2014, 374).

In comparison, some HWs used similar situations as a catalyst for reducing vulnerability for both the HC and themselves. For example, negotiating a re‐distribution of workload with colleagues to accommodate the extra time needed to manage private disclosures (Hersh 2015, 63), seeking out a trusted colleague to debrief with (Hersh 2015, 65), or avoiding the ‘open secret’ of drug use until the HC was ready to accept assistance for sobriety (Tiderington et al. 2013, 74).

These differences suggest HW responses to private disclosures have the potential to reduce vulnerability and stigma, and to increase healthcare safety and quality. Further research is needed to understand what strategies HWs find effective and what HWs need to implement these strategies to inform evidence‐based support and guidance for HWs managing private disclosures made to them by HCs.

### 4.1 Strengths and Limitations

This review constitutes part of a higher degree by research (HDR) qualification focusing on the Registered nurse perspective of receiving a private disclosure from an aged care resident. The use of broad search terms due to a lack of literature in this space greatly increased screening time. However, it became a strength representing the breadth and depth of the search, with results of interest informing the need for further research, policy review and education for nurses practicing within an interprofessional healthcare team. The inclusion of international literature made it possible to highlight cultural influence on what is considered a secret. The inclusion of relevant literature over a long‐time span has allowed authors to describe the impact of societal norms on secrets. The wide range of healthcare contexts documented in this scoping review suggests that private disclosure is relevant to all health settings, and that it can result in HW stress. Secrets associated with legal repercussions were not represented in Research Question 2 data; therefore, it was impossible to discuss the impact of secrets that invoke mandated reporting in this review.

In keeping with HDR process, data extraction was undertaken solely by Author 1 (KS). To reduce the risk of bias, a proportion of both included/excluded articles and extracted data were cross‐checked and approved by the supervisory team at the outset.

## 5 Conclusions

Substantial amounts of literature show that private disclosure is a common occurrence across a wide range of healthcare settings. The nature of the secrets revealed to HWs can be related to sexuality, medical concerns, abuse, and life choices and decisions. HWs think, feel and respond differently to private disclosures depending on their training, preparedness and relationship with the consumer. Their responses, in turn, have an influence on HC vulnerability and experience with health care. Given the lack of research on HW responses to disclosure, especially within the nursing profession, further research is required to provide guidance for health policy and support for HWs who find themselves as recipients of a private disclosure from an HC.

## Author Contributions

**Kiriaki Stewart:** conceptualization (equal); data curation (lead); formal analysis (lead); investigation (lead); methodology (equal); project administration (lead); validation (lead); visualization (equal); writing – original draft preparation (lead); and writing – review and editing (equal). **David Evans:** conceptualization (equal); data curation (supporting); formal analysis (supporting); investigation (supporting); methodology (equal); project administration (supporting); supervision (lead); validation (supporting); visualization (equal); writing – original draft preparation (supporting); and writing – review and editing (equal). **Qunyan Xu:** conceptualization (equal); data curation (supporting); formal analysis (supporting); investigation (supporting); methodology (equal); project administration (supporting); supervision (supporting); validation (supporting); visualization (equal); writing – original draft preparation (supporting); and writing – review and editing (equal). **Nicholas Procter:** conceptualization (equal); data curation (supporting); formal analysis (supporting); investigation (supporting); methodology (equal); project administration (supporting); supervision (lead); validation (supporting); visualization (equal); writing – original draft preparation (supporting); and writing – review and editing (equal).

## Conflicts of Interest

The authors declare no conflicts of interest.

## Supporting information


**Appendix S1.** PRISMA‐ScR checklist.

**Appendix S2.** Search strategy.

**Appendix S3.** Summary of articles for nature of secrets.

**Appendix S4.** Summary of articles for Healthcare worker experience.

## Data Availability

The data that supports the findings of this study are available in the Supporting Information of this article.
