# Supplementary material for: Secrets and Their Consequences in Heathcare: A Scoping Review of Worker Experiences
Source: J Adv Nurs. 2025 Mar 30;82(1):109–23. doi: 10.1111/jan.16922 (PMC12721927; doi:10.1111/jan.16922)
Supplement: Supplementary file 2 — Appendix S2. Search strategy. [file JAN-82-109-s001.docx]

**Supporting document 2: Search strategy**

**Database: Scopus** Most recently completed 4 November 2024 (2873 documents)

| Search within  Article title, Abstract, Keywords | Search documents  healthcare |
| --- | --- |
| AND | |
| Search within  Article title, Abstract, Keywords | Search documents  (consumer OR resident OR patient) AND (worker OR staff OR personnel OR employee) |
| AND | |
| Search within  Article title, Abstract, Keywords | Search documents  disclosure OR confession OR scandal OR secret OR private OR privacy |

Document type ***Limited to*** Article; Language ***Limited to*** English
