# Supplementary material for: Secrets and Their Consequences in Heathcare: A Scoping Review of Worker Experiences
Source: J Adv Nurs. 2025 Mar 30;82(1):109–23. doi: 10.1111/jan.16922 (PMC12721927; doi:10.1111/jan.16922)
Supplement: Supplementary file 3 — Appendix S3. Summary of articles for nature of secrets. [file JAN-82-109-s003.docx]

**Supporting document 3: Summary of articles for Nature of Secrets**

| **First author and year** | **Study design** | **Objective** | **Participants**  **And Number (n)** | **Concept (Secret)** | **Context** |  |
| --- | --- | --- | --- | --- | --- | --- |
| Agenor  2015 | Qualitative focus groups | To explore factors associated with cervical cancer screening. | Black LBQ women  n=18 | Sexual orientation: L / dating a woman. | Country – USA  Acute setting – ED  Community settings – CC, GC, GPC, OPD |  |
| Ait Gacem  2024 | Quantitative survey | To investigate CAM use disclosure to HPs. | HC  n=407 | CAM use | Country – UAE  Acute setting – H  Community settings –GPC, pharmacy |  |
| Algarin  2022 | Quantitative survey | To investigate the relationship between MSM disclosure experience to clinicians and rates of HIV testing, HBV or HPV vaccination. | HIV-ve MSM or unknown status MSM  n=13277 | Sexual behaviour: MSM | Country – Mexico  Healthcare setting – NR |  |
| Alvarez  2018 | Qualitative interview | To explore HW screening and response to IPV, and the use of a safety decision aid application (myPlanapp). | Medical doctors, doctors of osteopathic medicine, NPs, Midwives, Registered nurses, Social Workers, community healthcare workers  n=17 | IPV | Country – USA  Community settings – CC |  |
| Anguzu  2022 | Qualitative interview | To explore factors associated with perinatal IPV screening. | Enrolled midwife, Registered midwife, Medical Officer, Obstetrician, Gynaecologist, Assistant Nursing Officer  n=28 | IPV | Country – Uganda  Community settings – antenatal care clinic |  |
| Arrey  2015 | Qualitative interview | To explore factors associated with, experiences and management of HIV positive status disclosure. | Women PLWHA  n=28 | HIV status | Country – Belgium  Community settings – DC, GPC |  |
| Ashikaga  2002 | Quantitative survey | To investigate CAM use and communication with physicians in relation to lymphedema symptoms and other factors. | Women living with breast cancer  n=148 | CAM use | Country – Canada  Acute setting – H |  |
| Barbara  2001 | Qualitative interview | To explore factors associated with Lesbian HC sharing sexual orientation in a healthcare setting. | L  n=32 | Sexual orientation: L | Country – USA  Healthcare setting – NR |  |
| Bergman  2019 | Qualitative interview | To explore PCP’s care approach to women veterans with sexual trauma histories. | Doctors / NPs  n=28 | History of sexual trauma | Country – USA  Community setting – Veteran’ Health Administration clinic |  |
| Bernstein  2008 | Quantitative survey | To investigate the association between MSM disclosure of same-sex-attraction and self-reported HIV testing. | MSM  n=452 | Sexual orientation: GB / Sexual behaviour: MSM | Country – USA  Healthcare setting – NR |  |
| Bjorkman  2009 | Qualitative questionnaire | To explore the association between HC experiences and increased healthcare quality. | L  n=128 | Sexual orientation: L | Country – Norway  Community setting – GPC |  |
| Boehmer  2004 | | Qualitative interview | To explore Sexual orientation disclosure of LB women living with breast carcinoma. | LB women living with breast carcinoma  n=39 | Sexual orientation: LB | Country – USA  Acute setting – H  Healthcare setting – NR |
|  |  |  |  |  |  |  |
| Brinsdon  2017 | Qualitative interview | To explore Healthcare stigma experiences, management and reduction strategies of PLWHA. | PLWHA  n=44514 | HIV status | Country – NZ  Acute setting – H  Community settings – DC, optometry clinic |  |
| Cant  2006 | Qualitative interview | To explore the experiences of G men coming-out to general practitioners and sexual health clinic staff. | G men  n=38 | Sexual orientation: G | Country – UK  Community settings –GPC, SHC |  |
| Cao  2021 | Quantitative survey | To investigate the association between MSM disclosure to clinicians, rates of HIV testing, and antiretroviral prevention and PrEP therapy treatment. | MSM  n=689 | Sexual behaviour: MSM | Country – China  Acute setting – H  Community setting – clinic |  |
| Chang  2013 | Mixed method design | To understand predictors of CAM use disclosure to conventional HPs. | Outpatients  n=257 for survey / 39 for interviews | CAM use | Country – Taiwan  Community setting – OPD |  |
| Charbonnaeu  2010 | Quantitative survey | To investigate HIV status disclosure HC behaviours when seeking dental care. | PLWHA  n=453 | HIV status | Country – Canada  Community setting – DC |  |
| Chaudhary  2022 | Qualitative interview | To explore Transgender people’s experiences and expectations when interacting with pharmacists and pharmacy staff. | Transwomen, transmen, non-binary trans masculine  n=22 | Sexual orientation: Transgender | Country – Australia  Community setting – pharmacy |  |
| Cochran  1998 | Quantitative questionnaire | To investigate LB sexual orientation disclosure to physicians. | Black LB women  n=594 | Sexual orientation: LB | Country – USA  Healthcare setting – NR |  |
| Coleman  2017 | Quantitative survey | To investigate factors and implications associated with GBMSM disclosure to HPs, and health issues discussion. | GBMSM  n=173 | Sexual orientation: GB / Sexual behaviour: MSM | Country – Canada  Healthcare setting – NR |  |
| Currin  2017 | Quantitative survey | To investigate predictors of MSM disclosure. | GBMSM  n=148 | Sexual orientation: GB / Sexual behaviour: MSM | Country – USA  Healthcare setting – NR |  |
| Doran  2018 | Qualitative interview | To explore needs of GBMSM living with prostate cancer, and healthcare provision experiences. | GBMSM living with prostate cancer  n=12 | Sexual orientation: GB / Sexual behaviour: MSM | Country – UK  Community settings –SHC, UC |  |
| Durso  2012 | Qualitative interview | To explore predictors of LGB disclosure to HP. | LGB  n=396 | Sexual orientation: LGB | Country – USA  Healthcare setting – NR |  |
| Eliason  2001 | Qualitative questionnaire | To explore factors and prevalence of LGB disclosure, and frequency of protective strategies in healthcare settings. | LGB  n=88 | Sexual orientation: LGB | Country – USA  Healthcare setting – NR |  |
| Farooqui  2012 | Qualitative interview | To explore CAM use disclosure to HPs. | Cancer patients  n=12 | CAM use | Country – Malaysia  Acute setting – H |  |
| Fay  2011 | Quantitative survey | To investigate the association between discrimination, access to and use of HCs, HIV knowledge, and HIV prevalence. | MSM  n=537 | Sexual behaviour: MSM | Countries – Malawi, Namibia, Botswana  Healthcare setting – NR |  |
| Fish  2019 | Qualitative interview | To explore facilitators and barriers of LGB disclosure to HWs. | LGB living with cancer  n=30 | Sexual orientation: LGB | Country – UK  Healthcare setting – NR |  |
| Fuentes  2020 | Mixed method design | To explore understand motivations for and experiences of SMA. | Women who experienced SMA  n=721 for survey / 18 for interviews | SMA | Country – USA  Community setting – GC |  |
| Hersh  2015 | Qualitative interview | To explore traditional nursing ethos safeguards when caring for terminally ill hospitalized patients. | Palliative care nurses  n=17 | Decision to give children up for adoption (custody after the death of a parent). | Country – USA  Acute setting – acute care |  |
| Hinrichs  2018 | Focus groups | To explore PCP care improvements for TGNC patients. | TGNC  n=22 | Sexual orientation: TGNC | Country – USA  Community settings – GPC |  |
| Hitchcock  1992 | Qualitative questionnaire / interview | To explore factors associated with L disclosure to HPs, relationship management strategies, expected or experienced consequences, and health seeking and compliance behaviour. | L  n=33 | Sexual orientation: L | Country – USA  Healthcare setting – NR |  |
| Hudak  2015 | Qualitative interview | To explore HC navigation of LGB disclosure and existing communication barriers. | LGB  n=20 | Sexual orientation: LGB | Country – USA  Acute settings – ED, H  Community settings – clinics |  |
| Ismail  2018 | Qualitative interview | To explore CAM use disclosure to HPs. | Thalassaemia patients  n=21 | CAM use | Country – Malaysia  Acute setting – thalassaemia ward |  |
| Jansen  2024 | Quantitative survey | To investigate the association between medical mistrust and non-disclosure of kink-related injuries. | BDSM practitioners & Kink community  n=301 | Sexual behaviour: BDSM | Country – USA & Western Europe  Community setting – GPC |  |
| Jin  2022 | Qualitative interview | To explore the association between HP trust, health-seeking behaviours and health outcomes. | Chinese immigrants  Living with HBV  n=16 | CAM use | Country – Australia  Healthcare setting – NR |  |
| Johny  2017 | Quantitative survey | To investigate factors associated with CAM use disclosure among PCP attendees. | PCP attendees  n=906 | CAM use | Country – Malaysia  Community setting – GPC |  |
| Kamen  2015 | Quantitative survey | To investigate factors associated with LGBT living with cancer diagnosis, identity disclosure, and social support, and self-rated health. | LGBT living with cancer  n=291 | Sexual orientation: LGBT | Country – NR (USA-based online survey)  Healthcare setting – NR |  |
| Keefe-Oates  2022 | Qualitative interview | To explore factors associated with and experiences of SMA beyond 13 weeks with accompaniment group support, and HP interaction. | Women who experienced SMA beyond 13 weeks gestation  n= 23 | SMA | Country – Argentina  Acute settings – H  Community settings – radiology clinic, ambulance |  |
| Kelak  2018 | Qualitative interview | To explore the experience of CAM disclosure to doctor. | Primary Care clinic attendees  n=10 | CAM use | Country – Malaysia  Community setting – GPC |  |
| Lane  2008 | Qualitative interview | To explore MSM and HW interactions. | MSM  n=32 | Sexual behaviour: MSM | Country – South Africa  Community settings – clinics |  |
| Lasater  2019 | Quantitative survey | To investigate the association between migration and burden of stigma. | Female sex workers  n=354 | Sexual behaviour: Female sex worker | Country – Togo  Healthcare setting – NR |  |
| Lee  2020 | Quantitative survey | To investigate the association between immigrant status and MSM disclosure. | GBMSM  n=1010 | Sexual orientation: GB / Sexual behaviour: MSM | Country – USA  Healthcare setting – NR |  |
| Lewis  2019 | Quantitative survey | To investigate TGNC worries and coping actions related to HP discrimination. | TGNC  n=313 | Sexual orientation: TGNC | Country – USA  Community setting – Pharmacies |  |
| McCrea  2011 | Quantitative survey | To investigate HC disclosure and prevalence of concomitant mood-altering herb and prescription drug use. | College students  n=305 | CAM use | Country – USA  Healthcare setting – NR |  |
| Macapagal  2016 | Quantitative survey | To investigate LGBTQ emerging adult healthcare challenges. | LGBTQ  n=206 | Sexual orientation: LGBTQ | Country – USA  Healthcare setting – NR |  |
| Metheny  2016 | Quantitative survey | To investigate the association between MSM disclosure and uptake of routine HIV testing and HAV/HBV vaccination. | rural MSM  n=319 | Sexual behaviour: MSM | Country – USA  Healthcare setting – NR |  |
| Mimiaga  2007 | Mixed method design | To understand the association between MSM sexually transmitted diseases and HIV screening. | GBMSM  n=50 | Sexual orientation: GB / Sexual behaviour: MSM | Country – USA  Healthcare setting – NR |  |
| Morris  2025 | Mixed method design | To understand facilitators and barriers of shared decision making relating to IPV. | LGBTQ people of colour  n=217 | IPV | Country – USA  Healthcare setting – NR |  |
| Mosack  2013 | Quantitative survey | To investigate differences between sexual minority and heterosexual women healthcare experiences. | Sexual minority women  n=420 | Sexual orientation: LGBTIQ+ | Country – USA  Community setting –GPC |  |
| Nadler  2022 | Qualitative interview | To explore HP collection and documentation of gender identity. | NPs, Physicians, physician assistants  n=25 | Sexual orientation: gender identity | Country – USA  Community setting – GC, GPC, internal medicine wards, UC |  |
| Oldendick  2000 | Quantitative interview | To investigate black minority, rural, and low-income population CAM use. | CAM users in South Carolina  n=1584 | CAM use | Country – USA  Healthcare setting – NR |  |
| Papadakaki  2014 | Qualitative focus groups | To explore general practitioner perceptions and practices in identification and management of victimised patients. | Doctors  n=18 | IPV | Country – Greece  Community setting –GPC |  |
| Petroll  2015 | Quantitative survey | To investigate the association between health insurance and GBMSM disclosure to PCPs. | GBMSM  n=722 | Sexual orientation: GB / Sexual behaviour: MSM | Country – USA  Healthcare setting – NR |  |
| Petroll  2011 | Quantitative survey | To investigate the association between PCP knowledge of patients' sexual orientation, demographics, provider-related factors, and appropriate recommendations of preventive and diagnostic HCs. | MSM  n=271 | Sexual behaviour: MSM | Country – USA  Community settings – multiple clinics |  |
| Petronio  2011 | Qualitative interview | To explore nurses’ experiences of disclosure predicaments. | Critical care and emergency nurses  n=11 | Infidelity | Country – USA  Acute settings – ED, ICU, oncology ward |  |
| Raifman  2016 | Quantitative survey | To investigate the association between PrEP awareness and HP or community-based organisation contact. | GBMSM  n=401 | Sexual orientation: GB / Sexual behaviour: MSM | Country – USA  Healthcare setting – NR |  |
| Rank  2012 | Quantitative questionnaire | To investigate targeted HPV vaccination strategies. HPV vaccine acceptability and sexual experience prior to disclosure of MSM to HP. | MSM  n=1041 | Sexual behaviour: MSM | Country – Canada  Healthcare setting – NR |  |
| Risher  2013 | Quantitative interview | To investigate the association of healthcare seeking fear and MSM disclosure. | MSM  n=323 | Sexual behaviour: MSM | Country – SA |  |
| Rose  2016 | Qualitative survey / interview | To explore GB men living with prostate cancer experiences of sexual communication. | GB men living with prostate cancer  n=145 for surveys / 53 for interviews | Sexual orientation: GB / Sexual behaviour: MSM | Country – Australia, NZ, UK, USA  Healthcare setting – NR |  |
| Rounds  2013 | Qualitative focus groups | To explore LGBTQ perceptions of HP behaviours. | LGBTQ  n=11 | Sexual orientation: LGBTQ | Country – USA.  Acute setting – hospital  Community setting – GC |  |
| Ruiseñor-Escudero  2019 | Quantitative survey | To investigate the prevalence of MSM disclosure. | MSM  n=683 | Sexual behaviour: MSM | Country –Togo (West Africa)  Healthcare setting – NR |  |
| Shires  2023 | Qualitative focus groups / interviews | To explore sexual and gender minority cancer survivors healthcare experiences. | Black / White SGM cancer survivors  n=16 | Sexual orientation: SGM | Country – USA.  Acute settings – H  Community settings – GPC |  |
| Simundic  2018 | Quantitative survey | To investigate the prevalence of CAM use. HC knowledge and awareness of CAM use and potential impact on laboratory test results. | European HCs  n=3600 | CAM use | Country – 18 European countries  Community care – pathology OD |  |
| Singh  2018 | Quantitative interview | To investigate the association between MSM disclosure, sexually transmitted disease screening and related vaccine acceptance. | GBMSM  n=817 | Sexual orientation: GB / Sexual behaviour: MSM | Country – USA.  Acute settings – any HS  Community settings – any HC |  |
| Smith  2017 | Qualitative focus groups | To explore the association between LGBT persons' healthcare experiences description and perceptions of best practice. | LGBT  n=26 | Sexual orientation: LBT | Country – USA  Acute settings – any HC  Community settings – any HC |  |
| Stablein  2015 | Qualitative interview | To explore GBMSM perceived role of EHR in general and for disclosure. | GBMSM  n=30 | Sexual orientation: GB / Sexual behaviour: MSM | Country – USA  Acute settings – H  Community settings – GPC, SHC |  |
| Thompson  2016 | Qualitative focus groups | To explore TGNC HC perspectives and experiences of privacy and sensitive disclosures in EHR and healthcare settings. | TGNC  n=30 | Sexual orientation: TGNC | Country – USA  Acute settings – H  Community settings – GPC, SHC |  |
| Tiderington  2013 | Qualitative interview / field observation | To explore harm reduction and the consumer-provider relationship. | Residents of a Housing First accommodation project  n=10  Case managers: peer specialists, nurses, Social Workers, Substance Abuse Counsellors  n=14 | Substance dependency | Country – USA  Residential setting – housing project |  |
| Turton  2014 | Cross-sectional study | To investigate Oral health care stigma and discrimination experiences of PLWHA. | PLWHA  n=435 | HIV status | Country – SA  Community settings – DC |  |
| Wang  2024 | Qualitative interview | To explore sexual and gender minority cancer survivor needs, challenges and experiences. | SGM cancer survivors  n=30 | Sexual orientation: SGM | Country – Taiwan  Acute settings – H  Community settings – GPC |  |
| Wells-Prado  2022 | Quantitative survey | To investigate the association between GBM disclosure and sexual side effect discussions between HP and GBM prostate cancer survivors, and to determine if disclosure is situational or a consistent characteristic in this population. | GBM prostate cancer survivors  n=193 | Sexual orientation: GB / Sexual behaviour: MSM | Country – USA  Healthcare setting – NR |  |
| White  1998 | Quantitative survey | To investigate L perceptions of medical and alternative HP communication. | LB  n=324 | Sexual orientation: Lesbian / bisexual | Country – USA  Healthcare setting – NR |  |
| Wiginton  2021 | Quantitative survey | To investigate the association between MSM disclosure and healthcare related stigma. | MSM  n=3040 | Sexual behaviour: MSM | Country –  5 Sub-Saharan African countries  Healthcare setting – NR |  |
| Willie  2023 | Qualitative focus groups / interviews | To explore components required to develop a trauma-informed PrEP implementation program. | Black cisgender women  n=37  Program director, nurse, Social Worker, PrEP navigator  n=7 | IPV | Country – USA  Community setting – CC |  |
| Wong 2006 | Qualitative focus groups | To explore concealing, stigma coping strategies, and exclusionary effects of older PLWHA when accessing healthcare. | PLWHA  n=7 | HIV status | Country –  Hong Kong  Acute setting – H  Community setting – non-government organisation |  |
| Yang  2019 | Quantitative survey | To investigate the association between GBMSM disclosure to an HP and PrEP awareness. | GBMSM  n=192 | Sexual orientation: GB / Sexual behaviour: MSM | Country – USA  Healthcare setting – NR |  |

**Key**

AIDS - acquired immunodeficiency syndrome

B - bisexual

CAM - complementary and alternative medicine

CC - community clinic

DC - dentistry clinic

ED - emergency department

EHR - electronic health records

GB – gay or bisexual

GBMSM – gay, bisexual or men who have sex with men

GC - gynaecology clinic

GPC - General practitioner clinic, including primary care practice, family practice, primary care clinic

H – hospital

HAV – hepatitis A virus

HBV - hepatitis B virus

HC – healthcare consumer

HP – healthcare provider

HS – healthcare service

HW – healthcare worker

HIV - human immunodeficiency virus

HPV - human papillomavirus

ICU - intensive care unit

IPV - interpersonal violence

L – lesbian

LB – lesbian or bisexual

LGB – lesbian, gay or bisexual

LGBT – lesbian, gay, bisexual or transgender

LGBTQ - lesbian, gay, bisexual, transgender or queer

LGBTIQ+ - lesbian, gay, bisexual, transgender, queer and any additional identity terms

LBQ - lesbian, bisexual, or queer

MSM - men who have sex with men

NZ - New Zealand

NR - not reported

NP - Nurse Practitioner

OPD - out-patient department

PCP - primary care provider

PLWHA - person living with HIV / AIDS

PrEP - pre-exposure prophylaxis

SA - South Africa

SGM – sexual and gender minority

SHC - sexual health clinic

SMA - self-managed abortion

TGNC- transgender / gender non-conforming

UC - urology clinic

UK - United Kingdom

USA - United States of America
