# Supplementary material for: Secrets and Their Consequences in Heathcare: A Scoping Review of Worker Experiences
Source: J Adv Nurs. 2025 Mar 30;82(1):109–23. doi: 10.1111/jan.16922 (PMC12721927; doi:10.1111/jan.16922)
Supplement: Supplementary file 4 — Appendix S4. Summary of articles for Healthcare worker experience. [file JAN-82-109-s002.docx]

**Supporting document 4: Summary of articles for Healthcare worker response**

| **First author and year** | **Study design** | **Objective** | **Participants**  **And Number (n)** | **Response: healthcare workers views of and response to disclosure experience** | **Response: healthcare worker strategies to manage disclosure experience** |
| --- | --- | --- | --- | --- | --- |
| Alvarez  2018 | Qualitative interview | To explore healthcare worker screening and response to interpersonal violence, and the use of a safety decision aid application (myPlanapp). | Medical doctors, doctors of osteopathic medicine, Nurse Practitioners, midwives, registered nurses, Social Workers, Community Healthcare Workers  n=17 | Burden  Privilege | Avoidance  Avoided sensitive conversation.  Referred healthcare consumer to another service.  Facilitation  Created a safe space for disclosure of interpersonal violence by not allowing partner back into clinic room.  Nurtured healthcare worker – healthcare consumer relationship.  Revisited interpersonal violence screening once relationship and trust established. |
| Anguzu  2022 | Qualitative interview | To explore factors associated with perinatal interpersonal violence screening. | Enrolled midwife, Registered midwife, Medical Officer, Obstetrician, Gynaecologist, Assistant Nursing Officer  n=28 | Burden  Privilege | Avoidance  Referred healthcare consumer to another organisation.  Facilitation  Nurtured healthcare worker – healthcare consumer relationship to build rapport and confidence for disclosure to occur. |
| Bergman  2019 | Qualitative interview | To explore primary care provider’s care approach to women veterans with sexual trauma histories | Doctors / nurse practitioners  n=28 | Burden | Avoidance  Referred healthcare consumer to another colleague or department.  Facilitation  Nurtured healthcare worker – healthcare consumer relationship.  Added buffers in schedule to allow extra care when needed.  Asked colleagues to assist with workload when more time was needed with a healthcare consumer. |
| Hersh  2015 | Qualitative interview | To explore traditional nursing ethos safeguards when caring for terminally ill hospitalized patients. | Palliative care nurses  n=17 | Privilege | Facilitation  Nurtured healthcare worker – healthcare consumer relationship.  Rostered themselves on a shift to facilitate disclosure when a healthcare consumer need for catharsis was identified.  Personal  Sought out a trusted colleague for confidential debrief. |
| Nadler  2022 | Qualitative interview | To explore healthcare provider collection and documentation of gender identity. | Nurse Practitioners, Physicians, Physician Assistants  n=25 | Burden | Avoidance  Avoided asking sensitive questions.  Facilitation  Asked sensitive questions in a routine manner.  Created digital documentation shortcuts to save time.  Neutral stance  Neither avoided or facilitated disclosure. |
| Papadakaki  2014 | Qualitative focus groups | To explore general practitioner perceptions and practices in identification and management of victimised patients. | Doctors  n=18 | Burden | Avoidance  Treated wounds while avoiding sensitive discussion of their cause.  Facilitation  Nurtured healthcare worker – healthcare consumer relationship. |
| Petronio  2011 | Qualitative interview | To explore nurses’ experiences of disclosure predicaments. | Critical care and emergency nurses  n=11 | Burden  Privilege | Avoidance  Avoided certain healthcare roles to reduce possibility of disclosure.  Facilitation  Created a safe place for disclosure to occur.  Nurtured healthcare worker – healthcare consumer relationship.  Personal  Sought out a trusted colleague for confidential debrief. |
| Tiderington  2013 | Qualitative interview / Field Observation | To explore harm reduction and the consumer-provider relationship. | Case managers: peer specialists, nurses, Social Workers, Substance Abuse Counsellors  n=14 | Privilege | Avoidance  Avoided addressing sensitive topics until healthcare consumer was ready to discuss and accept healthcare assistance.  Facilitation  Nurtured healthcare worker – healthcare consumer relationship. |
| Willie  2023 | Qualitative focus groups / interviews | To explore components required to develop a trauma-informed PrEP implementation program. | Program director, nurse, Social Worker, PrEP navigator  n=7 | Burden  Privilege | Avoidance  Did not routinely screen for interpersonal violence.  Facilitation  Nurtured healthcare worker – healthcare consumer relationship to build rapport and trust. |
